# Transoral percutaneous radiofrequency ablation with a steerable needle and cementoplasty under CBCT and infrared augmented reality navigation system guidance for the treatment of a C1 solitary plasmacytoma: A case report

**DOI:** 10.1016/j.radcr.2023.11.016

**Published:** 2023-12-15

**Authors:** Eliodoro Faiella, Matteo Pileri, Domiziana Santucci, Claudio Pusceddu, Davide Fior, Federica Riva, Chiara Tagliaferri, Lorenzo Paolo Moramarco, Bruno Beomonte Zobel, Rosario Francesco Grasso

**Affiliations:** a Unit of Radiology and Interventional Radiology, Fondazione Policlinico Universitario Campus Bio-Medico, Via Alvaro del Portillo, Rome, Italy; b Research Unit of Radiology, Department of Medicine and Surgery, Università Campus Bio-Medico di Roma, Via Alvaro del Portillo, Rome, Italy; c Department of Oncological and Interventional Radiology, Businco Hospital, Cagliari, Italy; d Department of Radiology, Sant'Anna Hospital, San Fermo della Battaglia, Como, Italy

**Keywords:** Solitary plasmacytoma, C1 Vertebral, Cementoplasty, RFA, Trans-oral, Steerable needle, SIRIO Infrared augmented reality navigation system

## Abstract

We report a case of a 40-year-old female with a solitary plasmacytoma of the right transverse apophysis of C1 who underwent combined transoral ablation using a curved steerable needle and cementoplasty under CBCT and infra-red augmented reality navigation system. An imaging work-up revealed an osteolytic lesion determining partial collapse of the right lateral mass of C1 and involving the vertebral foramen. After a biopsy, that revealed a solid tissue consistent with plasmacytoma, it was decided to proceed with radiation therapy. Subsequent PET-CT restaging scans showed residual tumors treated with a transoral percutaneous approach, combining ablation and cementoplasty. This report evaluates the benefits of this combined procedure and the transoral approach, focusing on the advantages of steerable devices and navigation systems.

## Introduction

Solitary plasmacytoma (SPB), a rare plasma cell dyscrasia, may present as a single mass of monoclonal plasma cells, either extramedullary or intraosseous, as single (SPB) or several lesions multiple myeloma (MM). SPB most often occurs in bone [1]. The management of SPB involves a multidisciplinary approach to achieve optimal outcomes.

Radiofrequency ablation (RFA) is indicated for osteolytic or osteolytic–osteoblastic lesions with minimal extra-osseous components. The value of RFA and cementoplasty has been considered as palliative interventional strategies by the CIRSE and National Comprehensive Cancer Network (NCCN) Practice Guidelines for Adult Cancer Pain [2,3]. RFA on bone lesions has proven successful for pain management in patients, not candidates for surgery and radiation therapy (RT) and not receive adequate relief from pharmacologic therapy [4]. When these lesions are located in difficult-to-access sites, a steerable needle can access them, ensuring a complete ablation area and adequate safety margins through a single access channel.

In this case report, we present a patient with an SPB of C1 who underwent a transoral combined procedure of RFA using a steerable needle and cementoplasty positioned under CBCT and SIRIO infrared navigation system [5,6].

## Case report

**Fig. 1:**
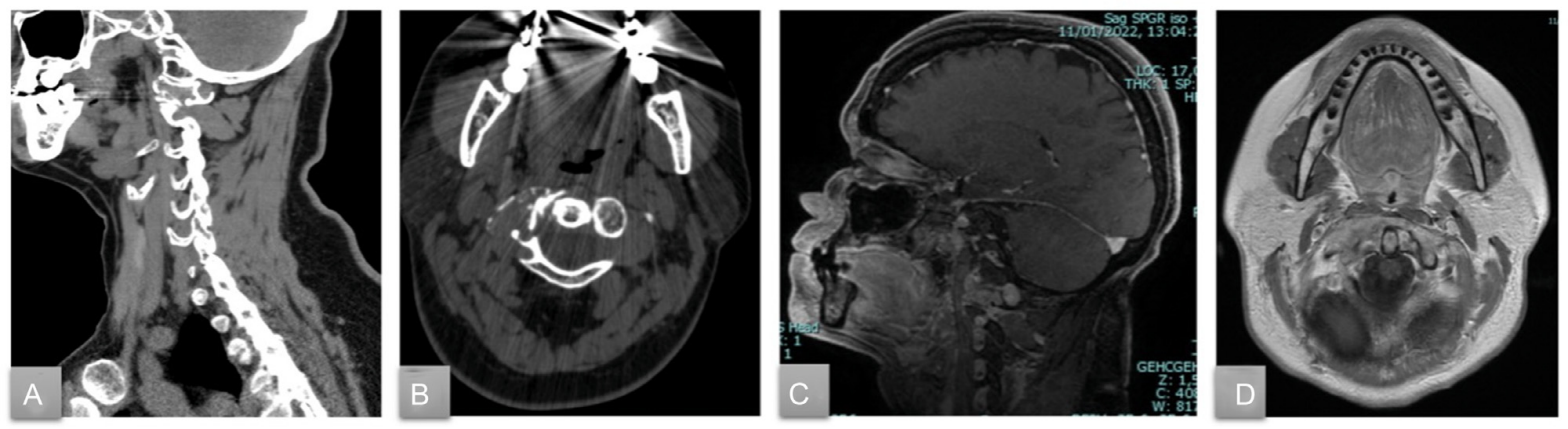
Preprocedural sagittal (A) and axial (B) CBCT scan showing the osteolytic lesion at the C1 vertebra. Preprocedural sagittal SPGR c+ (C) and axial T1 TSE c+ (D) MRI confirming the osteolytic lesion of C1 with heterogeneous postcontrast enhancement.

A 40-year-old patient was admitted in January 2022 to our department with progressively worsening and invalidating headaches in the last 3 months. The brain CT scan performed in the emergency department revealed an osteolytic lesion on the right lateral mass of the first cervical vertebra (C1), extending to the anterior arch and determining partial pathological collapse and complete obliteration of the right transverse foramen (Fig. 1). The subsequent MRI examination confirmed a lytic lesion involving C1 eroding cortical profiles with extension to the paravertebral tissues (Fig. 1). The patient was immobilized with a neck collar because of pain and the risk of fracture worsening.

The bioptic procedure was performed under general anesthesia through a transoral-endonasal approach with an endoscopic technique under CT guidance. At the histological examination SPB was diagnosed.

As disease staging confirmed the single lesion, it was initially decided to proceed with RT. The patient underwent a series of RT cycles targeting C1 from May to June 2022. A restaging PET scan and MRI performed in August 2022 revealed a partial response to the RT. Following a multidisciplinary evaluation, it was decided to proceed with a transoral percutaneous approach, combining ablation and cementoplasty.

**Fig. 2:**
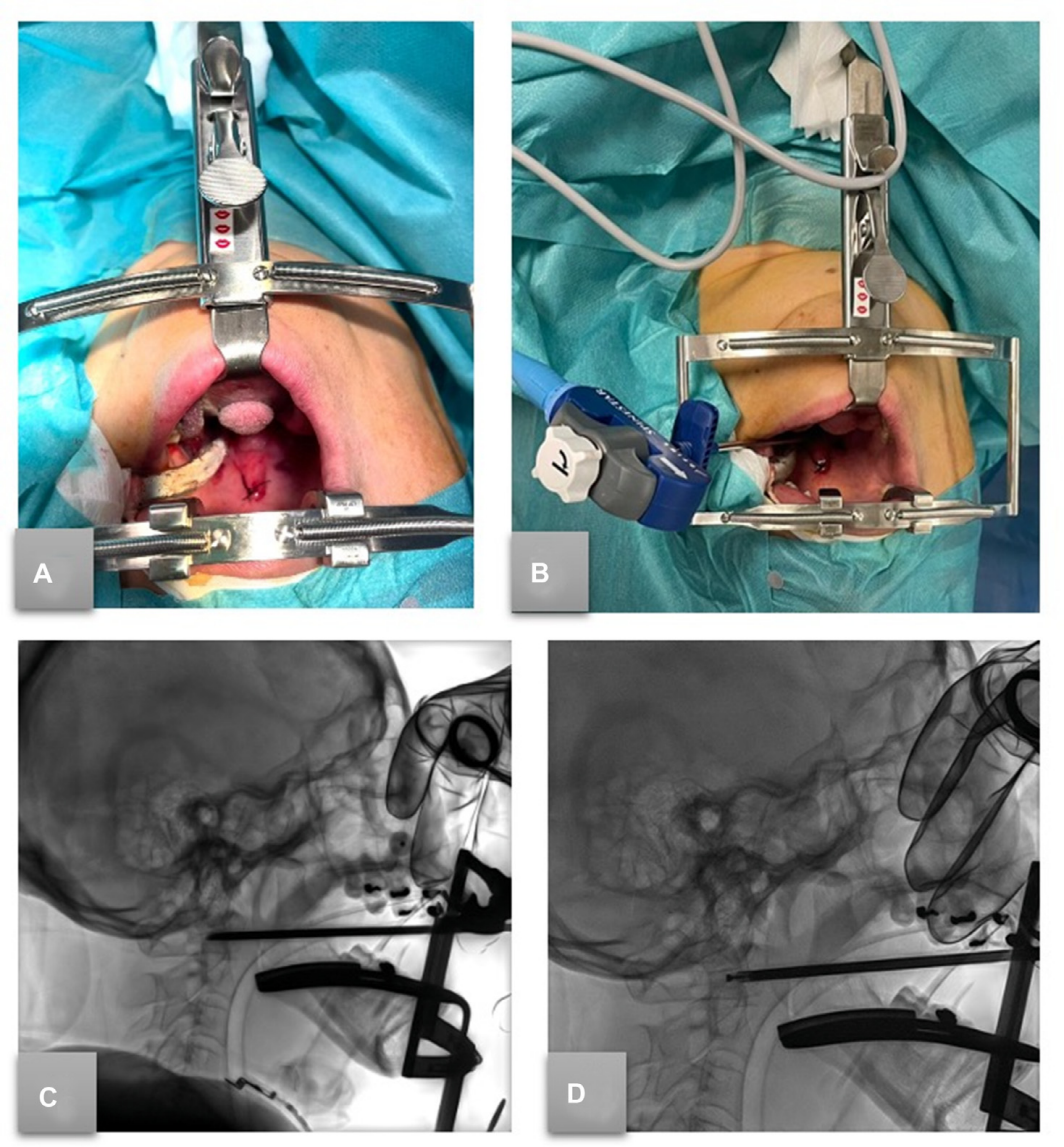
(A) Patient preparation: positioning of the dental retractor, and uvula retraction by suturing to expose the palate for needle access. (B) Intraprocedural RFA needle inserted transorally, in position before ablation. Sagittal fluoroscopic intraprocedural imaging of transoral access before (C, steerable osteotome transorally positioned) and after (D) the insertion of the RFA steerable STAR probe.

**Fig. 3:**
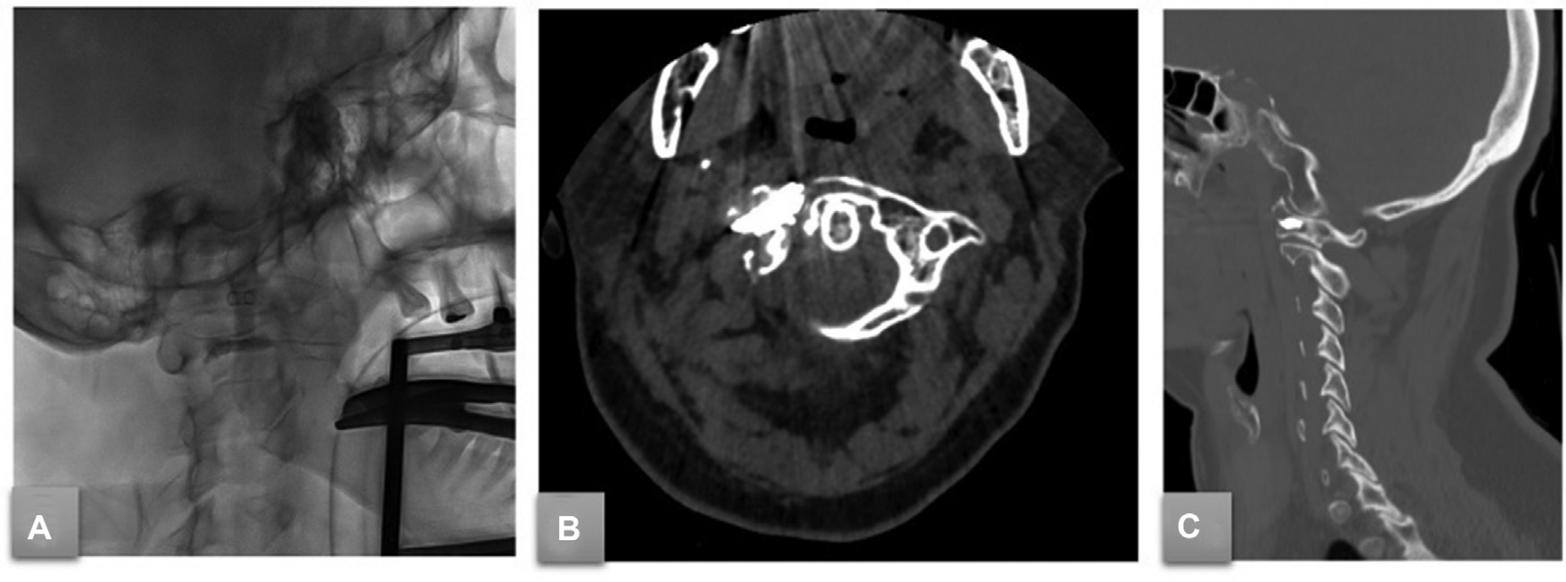
(A) Sagittal fluoroscopic post-procedural imaging after cementoplasty procedure. A 6-month postprocedural axial (B) and sagittal (C) CT scan after high-viscosity bone cement (StabiliT) injection.

Through CBCT guidance (Allura Xper FD20 Philips) with the assistance of an augmented reality infrared navigation system (SIRIO, MASMEC), it was possible to identify the bone abnormality and establish the desired trans-oral needle pathway. After dental retractor placement, the uvula retraction by suturing to expose the palate, and an IR performed the procedure under general anesthesia. The RFA procedure (Fig. 2) was conducted with the STAR Tumor Ablation System (STAR, Merit Medical Systems), including the steerable SpineSTAR ablation probe and the MetaSTAR generator. With the guidance of CT fluoroscopy, a 10G cannula was inserted through the oral mucosal tissue into the C1 right transverse apophysis, allowing the insertion of a steerable osteotome (PowerCURVE Navigating Osteotome) to create specialized channels for targeted ablation. Subsequently, the 11G RFA probe (SpineSTAR Ablation Instrument) was advanced coaxially through the cannula into the lesion site, enabling a focused ablation. Lastly, the cementoplasty was performed using the same cannula; a high-viscosity bone cement (STABILIT, Merit Medical Systems) was gradually delivered to ensure optimal filling of the ablated area (Fig. 3). The patient was asked to quantify her pain with the VAS score before and after (at 1 week, 3-,6- and 12-month) the treatment. Major and minor complications were evaluated based on the CIRSE classification system [2]. Local tumor control (LTC) was assessed with 6-month CE-CT scan and CE-MRI during the follow-up.

## Discussion

This case report describes a novel approach for treating a solitary plasmacytoma at the C1 vertebra using steerable devices and CBCT-navigation system guidance as tools for transoral RFA and cementoplasty.

As reported by Coleman et al. [7] in ESMO Clinical Practice Guidelines, several ablative therapeutic options are available for patients suffering from bone metastases, such as cryoablation, radiofrequency, and microwave ablation. The multidisciplinary team discussion is crucial to identify the correct clinical and therapeutic strategy for patients with single bone lesions.

Minimally invasive RFA treatment combined with cementoplasty provides a rapid and sustained long-term improvement in pain and quality of life, as reported in the literature [8] and confirmed in our report, demonstrating safety and quick pain relief.

The percutaneous approach to a bone lesion can be challenging based on the lesion's anatomical site and morphology [4]. In our case, the C1 lesion was highly challenging to approach posteriorly, therefore an anterior transoral approach was adopted.

Using a steerable needle under navigation system guidance allowed precise targeting and access to a difficult-to-reach lesion. This feature is due to the distal segment of the ablation probe that can be curved up to 90°, especially when treating tumors in challenging locations [9]. This approach enabled an anatomical adaptation to the oral cavity and the lesion morphology, guaranteeing complete ablation coverage and adequate safety margins through a single osseous access channel. The SIRIO system facilitated the identification of the correct needle pathway, reducing potential complications related to needle localization errors. This system allowed for the accurate positioning of the probe within the lesion, ensuring safety in ablative procedures [5]. Consequently, cementoplasty was performed using high-viscosity bone cement, injected into the ablated zone with a hydraulic system providing controlled delivery and minimizing potential complications.

Our patient showed a progressive decrease in pain and general relief, particularly during the 3-week follow-up visit. This is consistent with existing literature [10].

LTC was assessed through postprocedural imaging follow-up performed 1 month and 6 months after the procedure, showing no residual tumor or complications, confirming the postprocedural technical success. The patient removed the collar and discontinued pretreatment pain therapy 1 week after the procedure.

## Conclusion

The steerable device and navigation system combination constitutes a valuable tool for reaching complex sites and adapting the ablative area to the morphology of the lesion and the trajectory.

## Learning points

• Solitary plasmacytomas (SPB) often require a multidisciplinary approach for optimal management.
• Percutaneous thermal ablation, such as radiofrequency ablation (RFA), is an effective and minimally invasive treatment option for single-bone lesions.
• When bone lesions are located in difficult-to-access areas (ie, cervical vertebrae), using a steerable needle can provide safe access and ensure complete ablation coverage with adequate safety margins.
• The SIRIO navigation system enhances accuracy and precision in targeting and treating complex lesions, particularly in challenging anatomical locations.
• Combined transoral percutaneous RFA and cementoplasty can effectively treat solitary plasmacytomas, providing tumor ablation and stabilization of affected vertebrae.
• Long-term follow-up and further studies are needed to evaluate this combined approach's long-term efficacy and safety.
• Interventional Radiology techniques have a major impact on quality of life in patients affected by vertebral pathological fractures.

## Patient consent

Written informed consent was obtained from the patient for publication of this case report, including accompanying images.
